# The U.S.-Mexico Border Infectious Disease Surveillance Project: Establishing Binational Border Surveillance

**DOI:** 10.3201/eid0901.020047

**Published:** 2003-01

**Authors:** Michelle Weinberg, Stephen Waterman, Carlos Alvarez Lucas, Veronica Carrion Falcon, Pablo Kuri Morales, Luis Anaya Lopez, Chris Peter, Alejandro Escobar Gutiérrez, Ernesto Ramirez Gonzalez, Ana Flisser, Ralph Bryan, Enrique Navarro Valle, Alfonso Rodriguez, Gerardo Alvarez Hernandez, Cecilia Rosales, Javier Arias Ortiz, Michael Landen, Hugo Vilchis, Julie Rawlings, Francisco Lopez Leal, Luis Ortega, Elaine Flagg, Roberto Tapia Conyer, Martin Cetron, for the Border Infectious Disease Surveillance Project Working Group

**Affiliations:** * Centers for Disease Control and Prevention, Atlanta, Georgia, USA; †Secretaria de Salud, Mexico City, Mexico; ‡ San Diego County Health and Human Services Agency Public Health Laboratory, San Diego, California, USA; § Instituto de Diagnóstico y Referencias Epidemiológicas, Mexico City, Mexico; ¶ Universidad Nacional Autonoma de Mexico, Mexico City, Mexico; # Instituto de Servicios de Salud Publica de Baja California, Mexicali, Baja California, Mexico; ** California Department of Health Services, San Diego, California, USA; †† Secretaria de Salud Publica, Hermosillo, Sonora, Mexico; ‡‡ Arizona Department of Health Services, Tucson, Arizona, USA; §§ Servicios de Salud de Chihuahua, Chihuahua, Chihuahua, Mexico; ¶¶ New Mexico Department of Health, Santa Fe, New Mexico, USA; ## Border Epidemiology Center, Las Cruces, New Mexico, USA; *** Texas Department of Health, Austin, Texas, USA; ††† Servicios de Salud de Tamaulipas, Ciudad Victoria, Tamaulipas, Mexico; ‡‡‡ Pan American Health Organization, El Paso, Texas, USA

**Keywords:** border health, Mexico, southwestern United States, sentinel surveillance, communicable diseases, hepatitis, viral, human, migrant health, international health, infectious diseases, research

## Abstract

In 1997, the Centers for Disease Control and Prevention, the Mexican Secretariat of Health, and border health officials began the development of the Border Infectious Disease Surveillance (BIDS) project, a surveillance system for infectious diseases along the U.S.-Mexico border. During a 3-year period, a binational team implemented an active, sentinel surveillance system for hepatitis and febrile exanthems at 13 clinical sites. The network developed surveillance protocols, trained nine surveillance coordinators, established serologic testing at four Mexican border laboratories, and created agreements for data sharing and notification of selected diseases and outbreaks. BIDS facilitated investigations of dengue fever in Texas-Tamaulipas and measles in California–Baja California. BIDS demonstrates that a binational effort with local, state, and federal participation can create a regional surveillance system that crosses an international border. Reducing administrative, infrastructure, and political barriers to cross-border public health collaboration will enhance the effectiveness of disease prevention projects such as BIDS.

The 2,000-mile U.S.-Mexico border is one of the world’s busiest international boundaries. An estimated 320 million people cross the northbound border legally every year (*1*). The U.S.-Mexico border is a unique region where the geopolitical boundary does not inhibit social and economic interactions nor the transmission of infectious diseases among residents on each side of the border. Some border cities (such as El Paso and Ciudad Juarez) are separated by a short distance and serve as one large metropolitan area for the local community (Figure 1). From an epidemiologic perspective, the border population must be considered as one, rather than different populations on two sides of a border; pathogens do not recognize the geopolitical boundaries established by human beings. The border region has a population of approximately 11 million people (*2*), many of whom cross the border daily to work, shop, attend school, seek medical care, or visit family and friends (*3*,*4*). The border population also includes persons who pass transiently through the region and others who come the area to work in maquilas, the border factories. The region has experienced tremendous population growth. During 1993–1997, the U.S. border population grew by 1.8% annually, more than double the national U.S. average of 0.8%, while the Mexican border population has grown by 4.3% per year, almost three times the national Mexican annual growth rate of 1.6% (*2*,*5*). Population growth has been spurred by increased economic opportunities after the North American Free Trade Agreement was implemented in 1994. Currently, an estimated 3,300 maquilas, employing >1 million workers, are located along the border (*6*,*7*). The proliferation of border factories has generated a wave of internal migration of persons from other regions of Mexico and Central America toward the border (*8*).

**Figure 1:**
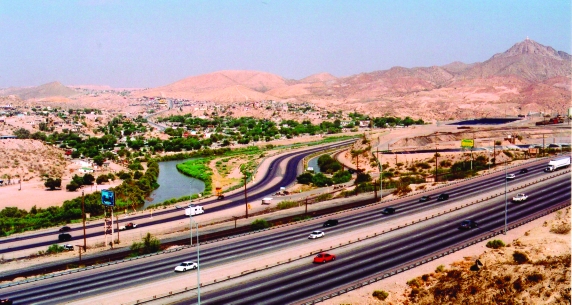
The Rio Grande River separates the border between Cuidad Juarez, Chihauhua, Mexico, and El Paso, Texas, USA.

From Mexico’s perspective, the border encompasses some of the country’s most economically prosperous states. In contrast, the U.S. border region is among the poorest areas in the United States, with >30% of families living at or below the poverty level (*8*). Along the Texas border, an estimated 350,000 or more people live in 1,450 unincorporated areas known as colonias, which lack adequate sanitation infrastructure (*8*).

The large population movement, limited public health infrastructure, and poor environmental conditions contribute to increased incidence of certain infectious diseases (*8*–*11*) Analysis of data from the U.S. National Notifiable Diseases Surveillance System for 1990 through 1998 showed increased risks for certain foodborne, waterborne, and vaccine-preventable diseases in U.S. counties within 100 kilometers of the border, compared with nonborder states. These data show a two- to fourfold greater incidence of hepatitis A, measles, rubella, shigellosis, and rabies and an eightfold greater incidence of brucellosis in border counties than in nonborder states (*11*). Studies have identified the importance of cross-border movement in the transmission of various diseases, including hepatitis A (*12*,*13*), tuberculosis (*14*–*18*), shigellosis (*19*), syphilis (*20*), *Mycobacterium bovis* infection (*21*), and brucellosis (*22*,*23*).

Despite the high prevalence of infectious diseases and increasing movement of people across the borders, no surveillance system had been established to assess the border population as a geographic unit. Gaining an accurate picture of public health needs was limited by the following factors. First, the surveillance case definitions used for public health reporting in Mexico and the United States are different. Also, laboratory confirmation is often unavailable in the Mexican border states, and therefore reported cases of infectious diseases are defined primarily by clinical findings. In contrast, for the many notifiable diseases in the United States, laboratory confirmation is required, and U.S. surveillance is heavily based on laboratory reporting. This system likely underestimates the true incidence rates. In the past, the two countries have exchanged limited border surveillance data. However, these differences diminish the usefulness of national surveillance data for developing a comprehensive, regional understanding of infectious disease epidemiology in the border areas. A consistent binational perspective is essential to effectively control and prevent the transmission of infectious diseases that move easily through the geopolitical boundary.

The Border Infectious Disease Surveillance (BIDS) project was designed to bridge this surveillance gap by forming partnerships among institutions in both countries serving the region and bringing together each country’s complementary experiences in syndromic and laboratory-based surveillance. This report describes the establishment of a binational surveillance system for hepatitis and febrile exanthems along the U.S.-Mexico border.

## Project Mandate

In June 1997, the United States–Mexico Border Health Association and the U.S. Council of State and Territorial Epidemiologists passed resolutions to support surveillance for infectious diseases and emerging infectious diseases along the U.S.-Mexico border (*24*,*25*). The Centers for Disease Control and Prevention (CDC) and the Mexican Secretariat of Health spearheaded efforts to initiate the project and formalized an agreement to establish BIDS through a memorandum of cooperation in epidemiology. A binational team of local, state, and federal epidemiologists, laboratory scientists, and public health officials met to organize and define project objectives. Decisions were made by consensus among the participants.

## Site Selection

**Figure 2:**
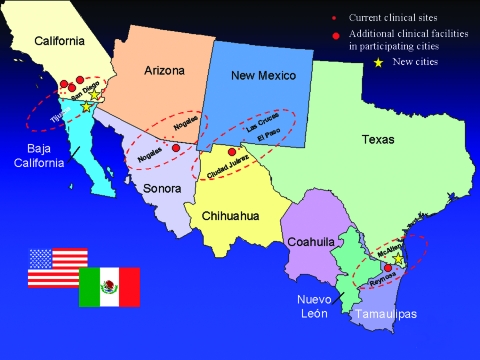
Border Infectious Disease Surveillance project sentinel sites in sister cities along U.S.-Mexico border: Tiajuana–San Diego, Nogales-Nogales, Las Cruces–Cuidad Juarez–El Paso, and Reynosa-McAllen. The new cities are Mexicali-Imperial (the sister city pair near Tijuana–San Diego) and Brownsville (near McAllen).

The team selected four sister city groups that had previously collaborated on binational projects (Figure 2). Local and state health departments identified one or more clinical facilities in each city. The U.S. institutions are four primary-care clinics and three tertiary care hospitals. The Mexican sites comprise two general hospitals and four primary-care clinics. The primary-care institutions service 10,000–20,000 acute-care visits per site annually, while the hospitals service 23,000–51,000 acute-care visits per site annually.

## Surveillance Strategy

After extensive discussions about local and national disease priorities, the team agreed on active sentinel surveillance for hepatitis and febrile exanthem syndromes. A standard protocol of laboratory testing is performed for specimens from patients who fulfill the clinical entry criteria (Table 1). Patients with acute hepatitis are tested at local laboratories for hepatitis A, B, and C. Depending on the initial results, specimens are tested at CDC for hepatitis D and E, and confirmatory testing for hepatitis C is performed. Patients with febrile exanthems are tested locally for measles and rubella. If these tests are negative, the specimens are tested at a state or national reference laboratory for rickettsiae, ehrlichiae, and, in selected areas, *dengue virus* (DENV).

**Table 1:** Entry criteria for active sentinel surveillance

Hepatitis	Illness with jaundice or dark urine, or illness >6 days without jaundice and >3 of the following: abdominal pain, alcoholic stools, nausea or vomiting, fever, anorexia
Febrile exanthem	Fever and nonvesicular rash, or an illness >3 days with fever but no rash, cough, and diarrhea

Many diseases selected for BIDS surveillance have well-defined preventive strategies, including vaccination. In Mexico, measles is targeted for elimination, and rubella vaccination was incorporated into the national vaccination program in 1998 (*26*). In the United States., rubella outbreaks have occurred among Hispanic immigrants who were not previously immunized (*27*–*30*). The south Texas border also represents an important zone for transmission of DENV (*31*–*33*) and typhus (*34*–*36*). In the United States, routine vaccination for hepatitis A was recently recommended for children in most of the U.S.-Mexico border region (*37*). Since laboratory confirmation was not readily available in Mexico, most acute hepatitis was assumed to be acute hepatitis A; however, the extent of hepatitis B and hepatitis C infection was unknown. Syndromic surveillance facilitated the monitoring of diseases targeted for elimination as well as identification of emerging infectious diseases, such as hepatitis E and ehrlichliosis, which have not been previously well studied along the border. The selection of these surveillance conditions was influenced by the need to establish laboratory infrastructure in Mexico; the hepatitis and febrile exanthem testing protocols involve serologic assays with similar equipment and assay techniques. From the practical perspective, sentinel surveillance enabled clinics in several border cities to participate at a reasonable cost. Active surveillance was selected to enhance existing passive surveillance activities in both countries, and, rather than creating a parallel structure, all activities were integrated into state and national reporting systems.

## Organizational Structure, Personnel, and Training

The group formed an executive committee and three subcommittees (epidemiology, laboratory, and communications). Nine sentinel site surveillance coordinators, who report to a local health department epidemiologist and a state-based epidemiologist, were hired and trained. The sentinel site coordinators are responsible for interviewing patients, completing data entry, and handling logistical issues such as specimen shipping and tracking. Considerable training was provided for border laboratory personnel. Only one of the Mexican border laboratories had experience performing serologic testing for hepatitis viruses, and none were experienced with testing for measle*s* or rubella viruses. Laboratory scientists from Instituto de Diagnóstico y Referencias Epidemiológicas, Mexico City, received training in testing methods for rickettsiae and ehrlichiae.

## Communications

Improving binational communication systems was critical to project success. In addition to language and cultural barriers, a large gap exists in communications infrastructure between the United States and Mexico. Many Mexican local and state health departments use combined telephone and fax lines and have no Internet access; regularly scheduled conference calls are a principal mechanism for communication. Borderwide meetings are held annually and sister city groups have regional meetings. A binational team of epidemiologists, laboratory scientists, and a representative from the El Paso Field Office of the Pan American Health Organization conducts site visits and evaluations.

## Logistics

The movement of equipment, supplies, specimens, and financial resources between the two countries has been difficult and labor-intensive. Logistical issues include the challenges of moving laboratory equipment and supplies into Mexico and specimens across the border into the United States. Several U.S. and Mexican agencies regulate these cross-border movements. Although regulations are established at a federal level, they are often subject to local interpretation. As a result, the BIDS participants work closely with local agencies in their state.

## Information-Sharing Protocols and Binational Cases

In the past, sharing of surveillance data has been constrained due to differences in political systems, limited forums in which to share information, and poor comparability of the data. Two protocols were developed to improve information sharing. The first describes the flow of information process in each country. Although the Mexican health sector is currently undergoing decentralization, officials from the federal Mexican Secretariat of Health continue to play the major role in reviewing, analyzing, and approving data before information is shared with U.S. counterparts and CDC. In contrast, U.S. local and state data can be shared without federal approval. The second protocol establishes conditions for urgent notification of sister city sites of outbreaks and cases of selected diseases, such as measles. Urgent notification also occurs for cases of binational public health importance, such as a case of hepatitis A in a person who works as a food handler on either side of the border. In the routine disease surveillance systems of both the United States and Mexico, a reportable condition diagnosed at a medical facility on one side of the border, in a patient who lives on the opposite side of the border, may not be included in routine disease reporting, and this information is not usually provided to the neighboring health officials. Data about these binational cases have not been traditionally captured in either country’s reporting system. Therefore, binational case definitions were developed for BIDS participants (Table 2).

**Table 2:** Case criteria for a binational case of hepatitis or a febrile exanthem

Binational case (at least one of the following):
1. Person with hepatitis or febrile exanthem who traveled or lived in neighboring country during incubation period for suspected or confirmed disease.
2. Person with hepatitis or febrile exanthem who had contact with persons who traveled or lived in neighboring country during incubation period for suspected or confirmed disease.
3. Case for which binational cooperation is needed for case investigation, case management, or both.

## Outcomes

The project timeline is shown in Table 3. Data collection began in late 1999, and data are currently being analyzed. As of mid-2002, the network had identified 867 persons with hepatitis and hepatitis syndrome (369 in the United States and 498 in Mexico) and 421 persons with a febrile exanthem syndrome (243 in the United States and 178 in Mexico). The project has enhanced local reporting of these conditions. In 2000, BIDS surveillance data from the sentinel site in Tijuana, Baja California, identified 300% more cases of laboratory-confirmed hepatitis A than would have been expected. All hepatitis A cases identified at the San Diego site were binational and were reported to the sister city health department in Tijuana.

**Table 3:** Timeline for implementation of Border Infectious Disease Surveillance project

1997	Mandate and objectives, site selection
1998	Binational planning, surveillance protocol with case definitions and data collection instruments
1999	Laboratory protocols and infrastructure; epidemiology training
2000–2001	Pilot data collection
2002–2003	Evaluation, consolidation, site expansion

The BIDS network has prompted valuable data exchange as well. In 2000, California and Baja California shared important information about measles in California and rubella in both states. The Baja California Health Department used BIDS surveillance data to conduct epidemiologic follow-up and targeted vaccination as part of their efforts to eliminate measles and reduce rubella. In 1999, the Mexican Secretariat of Health, CDC, and health officials from Texas and Tamaulipas conducted the first binational investigation of an outbreak of dengue fever (*38*).

## Future Plans

Consolidation, evaluation, and expansion of BIDS will take place in 2002–2003. The project, which began at 9 sites, has expanded to 13 sites. Continuing efforts will focus on incorporating six new clinical sites and three new border cities (Figure 2). With sufficient expansion, BIDS may be able to calculate population-based incidence rates for selected infectious diseases in some border areas. However, BIDS will continue to deal with the complexities of conducting surveillance among mobile populations and obtaining accurate denominator information for the border region.

Although activities focused initially on hepatitis and febrile exanthems, the project is sufficiently flexible to incorporate other syndromes and diseases, including *West Nile virus* and infectious agents that could be used in bioterrorism events. BIDS continues to improve communications systems and support mechanisms for information exchange.

## Conclusions

The BIDS project demonstrates that the development of a binational regional surveillance system for one of the world’s busiest geographic boundaries is feasible. Success is highly dependent on extensive U.S. and Mexican local and state involvement; maintaining a balance among the competing priorities of this diverse group of participants continues to be one of the project's greatest challenges. A high level of participation among the group is an essential ingredient in creating a binational, locally-relevant agenda and enhancing long-term project sustainability. The BIDS surveillance system has required flexibility to incorporate local and state reporting requirements, while maintaining sufficient standardization of case definitions, data, and laboratory testing procedures. Dedicated coordination at the federal level of both countries has been essential. Maintaining federal political commitment and funding for the 3-year development and implementation period has been and continues to be critical.

BIDS promotes communication and cooperation through border-wide meetings and the binational subcommittees. The border sister city meetings provide a forum for exchange of ideas and discussion of issues of binational importance, thus strengthening cross-border relationships among counterpart epidemiologists and laboratory staff. Many logistical problems require local solutions best handled by the sister cities working together. Effective problem solving requires coordination and optimal communication among participants. However, this level of effectiveness will not be achieved in the border region until the infrastructure barriers are overcome, including substantial improvements in access to telephones, fax machines, computers, the Internet, and satellite teleconferencing.

BIDS continues to face major obstacles in the movement of equipment, supplies, specimens, and financial resources across borders. These activities are cumbersome and time consuming for all project participants. Accords between the United States and Mexico should be developed to promote cooperation in public health and facilitate sharing of human and other resources and the moving of laboratory specimens across the border; these agreements would substantially enhance border health activities and benefit both countries. The states of Arizona and Sonora cooperated successfully in establishing a shared border health facility in Nogales, Sonora. This state-based model could be replicated in other areas of the border and reinforced with federal policies. To further enhance federal support, we suggest that a joint border field station be established by CDC, the Mexican General Directorate of Epidemiology, and the Instituto de Diagnóstico y Referencias Epidemiológicas.

The mandates from the United States–Mexico Border Health Association and the Council of State and Territorial Epidemiologists served as an initiation point for project activities but did not anticipate the need for additional federal-level agreements for data exchange. The BIDS group has drafted guidelines for conducting a binational outbreak investigation and will be implementing these as opportunities arise. Ongoing data collection will enable better characterization of binational cases. However, formal agreements at high levels of government are needed to authorize and endorse the timely binational exchange of epidemiologic and laboratory information about important infectious disease outbreaks and cases that occur along the border at BIDS sites and sites that are not currently part of the BIDS network.

Political changes at the local, state, and federal levels in Mexico frequently lead to changes in public health personnel, resulting in an ongoing need to train and incorporate new personnel into the project; this reality has highlighted the importance of institutionalizing any binational project through high-level formal agreements between the two countries. Weathering political changes is a challenge to infrastructure-building projects, like BIDS, which require several years of investment until tangible results, such as data are available.

To achieve the goal of building border epidemiology and laboratory capacity, BIDS established a system that built on existing strengths in syndromic and laboratory surveillance. The enhancement of border laboratory infrastructure at the Mexican sites was a major benefit. Additional support is essential, including stable funding for laboratory supplies and training courses, ranging from laboratory techniques to preventive maintenance of laboratory equipment; implementation of standardized quality control and quality assurance guidelines, such as a voluntary blinded proficiency testing program for selected tests; and a telephone consultation service with a toll-free telephone number to help staff address the problems.

Syndromic surveillance for hepatitis and febrile exanthems will allow us to estimate the magnitude of public health problems along the border, for example, acute hepatitis B and hepatitis C; determine the geographic distribution of diseases, such as typhus, ehrlichiosis, and dengue; detect outbreaks; evaluate control measures, such as immunization efforts to prevent measles, rubella, and hepatitis A and the reduction of breeding sites for mosquitoes that transmit dengue; and monitor emerging infections, such as hepatitis E, and generate hypotheses about these diseases that can be further studied. Systematic collection of surveillance data on binational cases will also better define the contribution of mobile populations to disease transmission.

As a model for true binational cooperation along the border, BIDS is a starting point from which a comprehensive infrastructure can be developed to accurately assess the health status of border residents and other migrants who come through the area. Ultimately, data provided by BIDS will be useful in the development of more effective prevention and control strategies for infectious diseases in this unique region.
